# Population-based nanopore sequencing of the HIV-1 pangenome to identify drug resistance mutations

**DOI:** 10.1038/s41598-024-63054-3

**Published:** 2024-05-27

**Authors:** Hirotaka Ode, Masakazu Matsuda, Urara Shigemi, Mikiko Mori, Yoshimi Yamamura, Yoshihiro Nakata, Reiko Okazaki, Mai Kubota, Yuka Setoyama, Mayumi Imahashi, Yoshiyuki Yokomaku, Yasumasa Iwatani

**Affiliations:** 1 Clinical Research Center, National Hospital Organization Nagoya Medical Center, 4-1-1 Sannomaru, Naka-ku, Nagoya, Aichi 460-0001 Japan; 2 Division of Basic Medicine, Nagoya University Graduate School of Medicine, Nagoya, Aichi Japan

**Keywords:** Nanopore sequencing, HIV-1, Drug resistance, Viral genome, Mutation, Recombinant forms, Pangenome, HIV infections, Genetics research, Medical research, Epidemiology, Viral genetics, Sequencing

## Abstract

HIV-1 drug resistance genotypic tests have primarily been performed by Sanger sequencing of gene segments encoding different drug target proteins. Since the number of targets has increased with the addition of a new class of antiretroviral drugs, a simple high-throughput system for assessing nucleotide sequences throughout the HIV-1 genome is required. Here, we developed a new solution using nanopore sequencing of viral pangenomes amplified by PCR. Benchmark tests using HIV-1 molecular clones demonstrated an accuracy of up to 99.9%. In addition, validation tests of our protocol in 106 clinical samples demonstrated high concordance of drug resistance and tropism genotypes (92.5% and 98.1%, respectively) between the nanopore sequencing-based results and archived clinical determinations made based on Sanger sequencing data. These results suggest that our new approach will be a powerful solution for the comprehensive survey of HIV-1 drug resistance mutations in clinical settings.

## Introduction

HIV-1 exhibits remarkably high genetic diversity resulting from its high mutation and recombination rates during viral replication. This diversity makes it challenging to analyze HIV-1 genome sequences. HIV-1 is classified into four genetic groups (M, N, O and P). Group M, which is the globally dominant epidemic variant, can be further subdivided into ten subtypes (A, B, C, D, F, G, H, J, K and L) and their recombinant forms (RFs)^1,2^. The nucleotide divergences of HIV-1 genomes between different subtypes and between variants in a subtype are approximately 15% and 8%, respectively^3^. According to the Los Alamos HIV sequence database (https://www.hiv.lanl.gov/), an increasing number of circulating RFs (CRFs) have been identified^1,2,4^. Information on the genetic diversity of HIV-1 sequences is important for viral load determination, antiviral drug development and epidemiological estimation of HIV-1 transmission in populations.

In addition, sequencing analyses of HIV-1 genes are employed for routine drug resistance (DR) tests to select appropriate drugs and consequently prevent the emergence of DR^5^. Because intrahost viral populations usually consist of quasispecies bearing various divergent sequences, dominant as well as minor populations are evaluated for DR in the present tests. To date, Sanger sequencing has typically been used for the analysis of particular viral protein-coding sequences (hereafter referred to as vCDS) encoding drug targets. This sequencing approach can generally identify low-abundance mutations with prevalences of 15–25% or greater. In contrast, deep sequencing approaches based on Illumina or Ion Torrent technologies that have recently been developed for DR testing are able to detect less abundant mutations (~ 1%), although the clinical impact of detecting such low-abundance mutations remains controversial^6,7^. Currently, the nucleotide sequence of *pol*, which encodes three viral gene products—protease (PR), reverse transcriptase (RT) and integrase (IN)—is routinely examined prior to antiretroviral therapy (ART) to determine the suitability of ART regimens containing PR inhibitors (PIs), nucleoside analog RT inhibitors (NRTIs)/nonnucleoside RT inhibitors (NNRTIs) and/or IN strand transfer inhibitors (INSTIs)^8,9^. Optionally, a partial *env* sequence encoding gp120 V3 can be examined to determine susceptibility to the virus entry inhibitor maraviroc (MVC). Moreover, it is desirable to examine the capsid (CA) sequence in the HIV-1 *gag* region to assess suitability for treatment with the first-in-class CA inhibitor lenacapavir (LEN), which has recently been approved for treatment-experienced patients with multidrug-resistant HIV-1^10^. Hence, HIV-1 DR testing requires the analysis of more multiple-segmented genes in the HIV-1 genome than was previously necessary.

One alternative approach to Sanger sequencing is nanopore sequencing (Oxford Nanopore Technologies [ONT]), which provides sequence information for long DNA fragments (tens to hundreds of kilobases) within short periods^11^. The system is cost-efficient and portable/accessible, even in countries where resources are limited but the burden of HIV is high. We previously reported that a nanopore sequencing-based approach was able to identify the near-full-length HIV-1 genome structure of intersubtype recombinant HIV-1 and dual-infection heterologous HIV-1 despite a high error rate within the raw reads (~ 6.5%)^4^. The approach was based on a previous flow cell (R9.4.1) and its compatible chemistry (V10) with the simplex (single-strand) base-calling program. Recently, ONT has released newer versions of flow cell (R10 series) and new chemistries (e.g., V12 and V14) with improved accuracy for simplex reads^12^ as well as the “duplex” method. The duplex method allows the sequences of both strands of each DNA molecule to be determined in succession and consequently provides a set of “duplex” reads of the double-stranded DNA molecule, with greater accuracy than the simplex method. In this study, we developed a new approach for HIV-1 DR genotyping using the latest version of nanopore sequencing technology for determination of the near-full-length HIV-1 genome. Validation with clinical samples demonstrated that the DR test outcomes obtained by our protocol were concordant with archived Sanger sequencing-based data. This solution could be implemented in clinical settings globally as a simpler and more comprehensive DR test, even for resource-limited settings.

## Results

### Assessment of nanopore sequencing error rates for the HIV-1 genome

**Figure 1:**
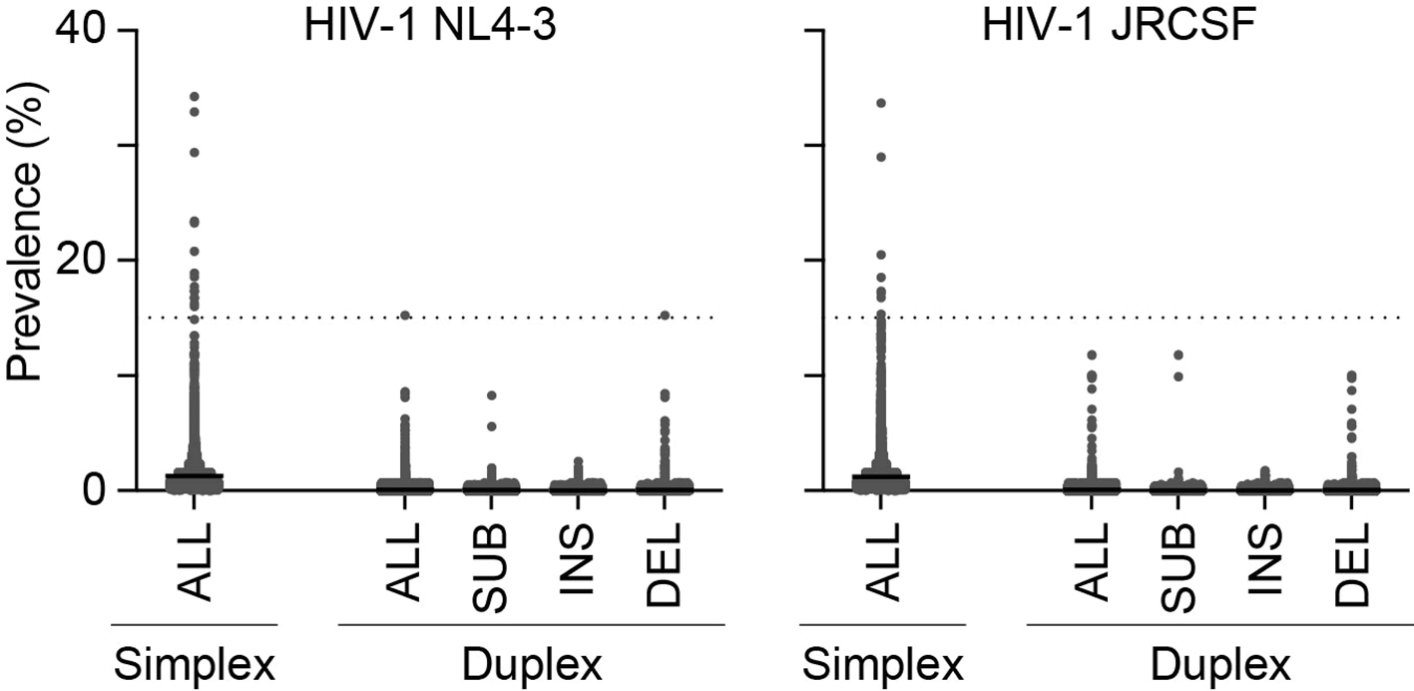
Prevalence of errors at each nucleotide position within the entire viral protein-coding sequence (vCDS) from simplex and duplex reads. The nucleotide sequences were determined by nanopore sequencing using a mixture of three independent amplicons for HIV-1 NL4-3 or HIV-1 JRCSF propagated in cell cultures. The prevalence (%) of all mutations (ALL), substitutions (SUB), insertions (INS) and deletions (DEL) at each position in mixture from three is plotted. The horizontal dotted lines highlight the 15% prevalence threshold.

A new nanopore sequencing-based approach based on our previous system^4^ with slight modifications was constructed to obtain information on near-full-length HIV-1 genomes. Through this approach, we first compared the error rates between simplex and duplex reads using samples of HIV-1 NL4-3 and HIV-1 JRCSF that were propagated in cell cultures. To minimize the possibility to detect PCR-induced errors, a mixture of three independent amplicons were subjected to sequencings as previously reported^13,14^. In this study, the errors are defined as mutations including substitutions, insertions and/or deletions in reads by comparing reads with their corresponding sequences of HIV-1 NL4-3 and HIV-1 JRCSF molecular clones. The results demonstrated that the duplex reads are > tenfold more accurate than the simplex reads (Fig. 1). The mean prevalence of the errors in duplex reads reached 0.089% and 0.078% for NL4-3 and JRCSF. When the prevalence (%) of errors in the duplex reads was scored at each position of the entire vCDS, we found that deletions were more prevalent than substitutions or insertions (Fig. 1, p < 0.0001 between the deletions and insertions, and *p* = 0.23 and 0.01 between the deletions and substitutions for NL4-3 and JRCSF, respectively). Similar to a previous report^12^, these deletion errors were most commonly observed at homopolymeric stretches. For example, we observed a single nucleotide deletion at a stretch of six A bases within the *env* (position 7750 nucleotide [nt] in the HXB2 reference) with high prevalence in both NL4-3 (15.2%) and JRCSF (10.0%) and another single-nucleotide deletion at a six-A bases within *pol* (position 2849 nt) with an error prevalence of 9.9% in JRCSF (Fig. 1). In contrast, all substitution errors were present in < 15% of the population and insertions were much less prevalent than substitutions (*p* < 0.0001). Therefore, we hereafter focused on duplex reads and set 15% as the threshold for the detection of minority molecule populations. Moreover, we required ≥ 100 × coverage of duplex reads comprising the entire vCDS for each sample to ensure the detection of mutations present in 15% of the populations.

### Nanopore sequencing of the HIV-1 pangenome in clinical samples

**Figure 2:**
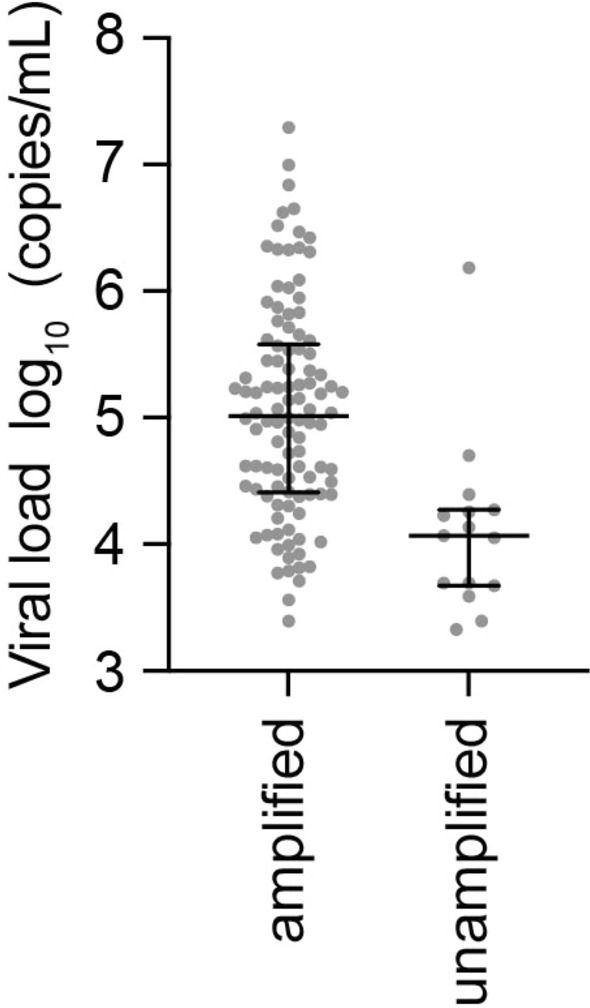
Success and failure rates of HIV-1 pangenome amplification via RT–PCR and nested PCR for clinical samples (n = 121) with different viral loads. The log10-transformed viral loads are plotted with gray dots in two groups: successful amplification (amplified) and no amplification in any of the quadruplicate reactions (unamplified). The median and interquartile range (IQR) are shown with black bars.

We evaluated our protocol for nanopore sequencing-based DR tests using 121 archived clinical samples obtained from treatment-naïve patients that had also been subjected to Sanger sequencing-based test between 2020 and 2023 in our hospital. These samples represented patients with a wide range of viral loads (between 2120 and 19,700,000 copies/mL; median 88,300; interquartile range [IQR] 18,350–332,550) and CD4^+^ T-cell counts (between 1 and 1022 cells/µL; median 186; IQR 46–342). Among the 121 samples, viral pangenomic DNA libraries derived from multiple amplicons were successfully prepared for 106 samples, which were associated with relatively greater viral loads (median 103,550; IQR 25,825–380,250) than the 15 samples for which pangenomic DNA amplifications failed (median viral load 11,700 copies/mL; IQR 4700–18,700) (Fig. 2). After obtaining the entire vCDS duplex reads by nanopore sequencing for the 106 clinical samples, we determined the HIV-1 subtype of each sample through the jpHMM-HIV^15^. The results showed that the subtypes determined using nanopore sequencing-based information were perfectly consistent with those determined according to the archived Sanger sequencing data. Among the 106 samples, the dominant subtypes were subtype B (73.6%), followed by CRF01_AE (AE) (10.4%), subtype C (3.8%) and unique RFs (URFs) (12.3%). No sample displayed any mixed heterologous subtypes. This subtype trend is similar to the recent epidemic report throughout Japan^16^.

**Figure 3:**
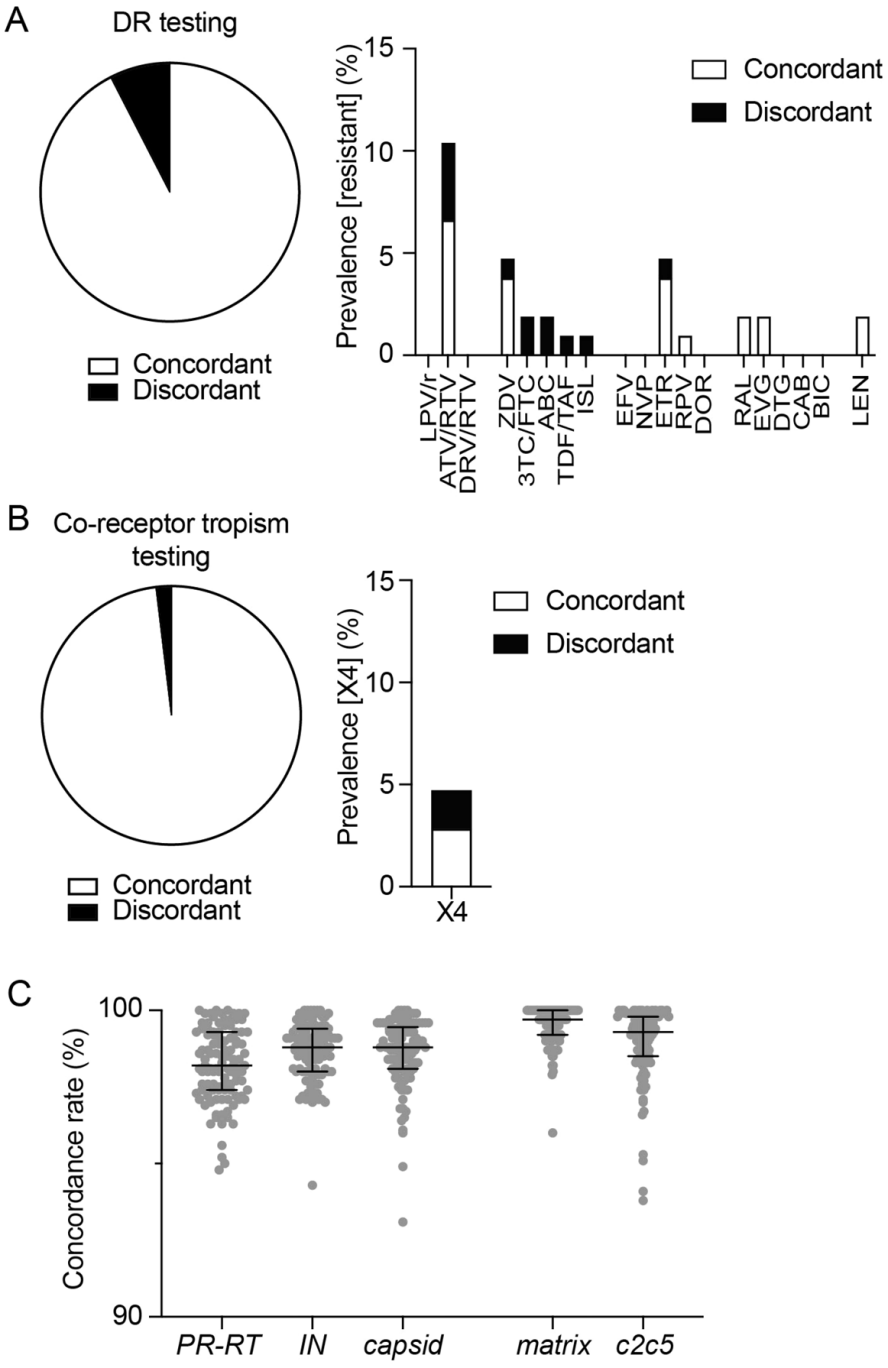
Comparisons of nanopore sequencing DR data with archive DR test data obtained by Sanger sequencing. (**A**) Concordance rates (%) of the DR tests between Sanger and nanopore sequencing. The prevalence of detected DR to each drug is displayed with bar graphs. The “Concordant” and “Discordant” columns represent fully matched and inconsistent results between the two sequencing methods. LPV/r: lopinavir boosted with ritonavir, ATV: atazanavir, DRV: darunavir, RTV: ritonavir, ZDV: zidovudine, 3TC: lamivudine, FTC: emtricitabine, ABC: abacavir, TDF: tenofovir, TAF: tenofovir alafenamide, ISL: islatravir, EFV: efavirenz, NVP: nevirapine, ETR: etravirine, RPV: rilpivirine, DOR: doravirine, RAL: raltegravir, EVG: elvitegravir, DTG: dolutegravir, CAB: cabotegravir, BIC: bictegravir, LEN: lenacapavir. (**B**) Concordance rates of coreceptor tropism results based on V3 sequences between the two sequencing methods. The concordance rates of CXR4 (X4) tropism are displayed with a bar graph. Putative tropisms were determined by geno2pheno-C_NGS-Sanger (https://coreceptor.geno2pheno.org/). (**C**) The concordance rates of nucleotide sequences in the *pol PR-RT*, *pol IN, gag capsid, matrix* and *env c2c5* regions are plotted for each sample with medians and interquartile ranges.

Next, we compared the DR test outcomes between the nanopore and Sanger sequencing methods. The genotypes per nucleotide position were classified as “resistant”, “possibly resistant” or “susceptible” according to the presence or absence of the defined mutation sets in the ANRS V34 algorithm (November 2023) (https://hivfrenchresistance.org/). Here, we define “fixed” and “nonfixed” mutations as mutations found in > 90% and 15–90% of the population of nanopore sequencing reads, respectively. For Sanger sequencing-based DR test results based on *pol PR-RT* (2253–3269 nt) or *pol IN* (4230–5093 nt), mixed bases at heterogeneous peaks were manually picked from electropherogram data^4,17–19^. As shown in Fig. 3A, the overall concordance rate of the DR test results was 92.5% for all of the drugs listed, including the three PIs, the five NRTIs, the five NNRTIs, and the five INSTIs. DR mutations to zidovudine (ZDV) (RT T215D/E/S) and rilpivirine (RPV) (RT E138A) were consistently observed for four and one samples, respectively, in the data from both methods. The IN E138Q mutation associated with resistance to raltegravir (RAL) and elvitegravir (EVG) was concordantly detected in two samples (Fig. 3A). In addition, possible DR mutations to atazanavir/ritonavir (ATV/RTV) and etravirine (ETR) were detected in seven and four samples, respectively. In contrast, discordant DR test data for ATV/RTV, ZDV, lamivudine/emtricitabine (3TC/FTC), abacavir (ABC), tenofovir/ tenofovir alafenamide (TDF/TAF), islatravir (ISL), and ETR were found in 8 samples (7.5%). Importantly, the discordant results were due solely to inconsistent detection of a single, nonfixed mutation for each of the 8 samples, and no differences were observed in the detection of fixed mutations that are associated with resistance or possible resistance to any drugs. Single mutations in mixed populations (PR L10F, PR G16E, PR D62E, RT K65R, or RT V106I) were found in 5 patients by Sanger sequencing, although these mutations were not identified by nanopore sequencing. In contrast, nonfixed mutations (PR M46I [17.8%], RT M184I [23.5%] or RT T215S [20.3%]) were detected in only three patients with nanopore sequencing-based results. These results indicate that the difference between the two methods was largely due to the probability of distinct amplicon amplification by PCR. Moreover, we examined fixed and nonfixed amino acids at the 72 DR-associated positions listed in the ANRS algorithm tables. The results showed that medians of the positions where concordant and discordant amino acid mutations relative to the HXB2 reference were identified were 3 (IQR 2–3) and 0 (IQR 0–1), respectively. The concordance rate of amino acids per DR-associated position was 99.1%. These results suggest that nanopore sequencing-based DR testing is comparable to Sanger sequencing-based DR testing.

Genotypic coreceptor tropism, that is, CCR5 (R5) or CXCR4 (X4) usage, was also examined based on V3 amino acid sequences using the geno2pheno-C_NGS-Sanger tool^20^ (https://coreceptor.geno2pheno.org/). For the nanopore sequencing data, sequence contexts present in ≥ 15% of the population in each sample were used, while for the canonical test using Sanger sequencing data, nucleotide sequences of the *env c2c5* gene (6896–7652 nt) from three independent amplicons for each sample were analyzed to identify low-abundance variants within each sample at the most heterogeneous *env*^4,17–19^. We observed high concordance (98.1%) between two methods for both genotypic tropism tests (Fig. 3B). Two cases of discordant X4 detection were observed in the nanopore sequencing datasets, largely because a large amount of sequence contexts were obtained by nanopore sequencing, whereas only three major nucleotide sequences were extracted from Sanger sequencing data. Therefore, the sequences from Sanger sequencing data might not contain sequences of minority variants.

We further compared the concordance rates of entire consensus nucleotide sequences in the *pol PR-RT*, *pol IN*, *gag matrix* (790–1185 nt) and *env c2c5* regions between the two sequencing methods. Mixed bases are included in the sequences of *pol PR-RT* and *pol IN*, but not in those of the *gag matrix* and *env c2c5* because high heterogeneity of the *matrix* and *env c2v5* makes it difficult to identify low-abundance mutations^3^. The concordance rates for each sample were 98.2% (IQR 97.4–99.3%), 98.8% (IQR 98.0–99.4%), 99.7% (IQR 99.2–100.0%) and 99.3% (IQR 98.5–99.8%) for *pol PR-RT*, *pol IN*, *gag matrix* and *env c2c5*, respectively (Fig. 3C). The results suggest that consensus nucleotide sequences are also highly concordant between nanopore and Sanger sequencing for clinical samples. In addition, the minor discordance between the two methods may be due to amplification bias by RT-PCR/nested PCR or different sequencing read depths, as observed in the DR tests.

The *gag capsid* (1186–1878 nt) sequences, which are associated with LEN-associated DR mutations, were also analyzed. The consensus *gag capsid* sequences containing mixed bases determined by Sanger sequencing (“Supplementary Methods”) were compared with the nanopore sequencing data of 106 samples. The samples examined in this study were obtained from treatment-naïve patients before LEN was approved in Japan. The results represented polymorphic mutations, CA T107A or CA T107S, found in two cases by two methods, which do not impact LEN susceptibility but emerge with primary LEN-associated DR mutations^21,22^. The rare detection of LEN-associated DR mutations before LEN approval is consistent with a previous report^23^. The median concordance rate per nucleotide was 98.8% (IQR 98.1–99.5%) for the two methods (Fig. 3C), which is comparable to that for *pol PR-RT* and *pol IN*. In addition, when we analyzed amino acids at the 7 positions associated with LEN resistance, the results represented 99.9% of concordant amino acids at the positions. These results suggest that nanopore sequencing-based DR tests could also be applied to the *gag capsid* gene.

### Intrasample viral genetic diversity

**Figure 4:**
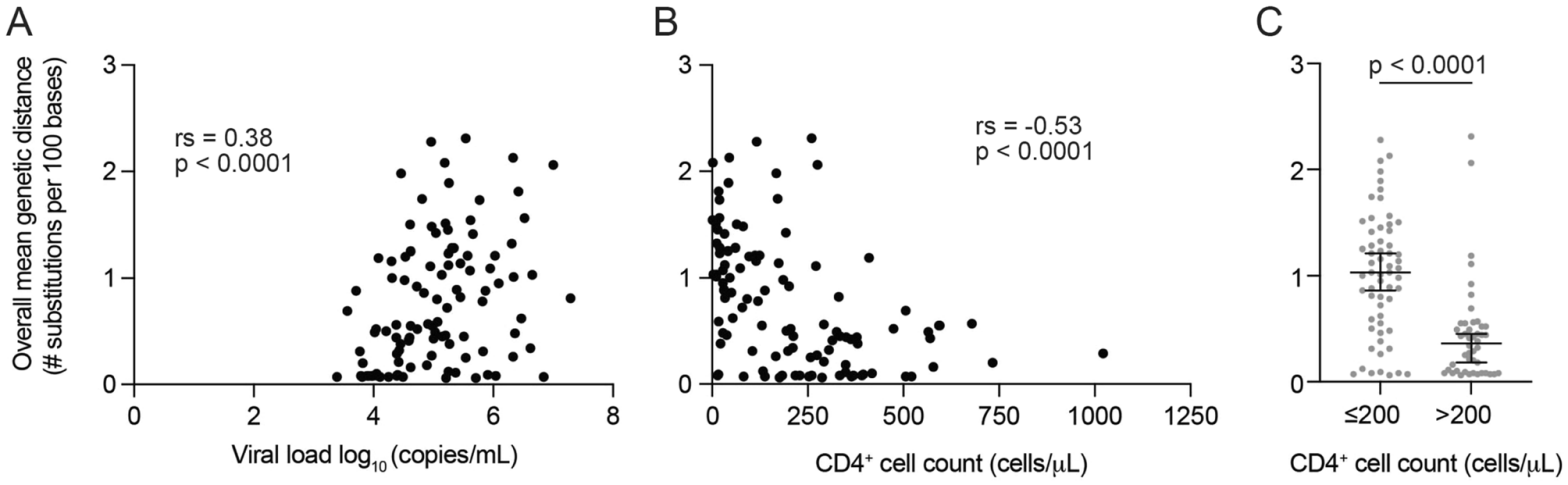
Relationship of intrasample genetic diversity with two surrogate markers of HIV-1 disease progression: viral load and CD4^+^ T-cell count. The genetic diversity was assessed based on the overall mean genetic distances between every pair of complete viral protein-coding sequences (vCDSs) for each sample. Correlations of genetic diversity with viral load (**A**) or CD4^+^ T-cell count (**B**) were evaluated with the nonparametric Spearman’s rank-order test. *C*, genetic diversities were compared between the samples with CD4^+^ T-cell counts of ≤ 200 and > 200 cells/μL. Statistical significance was tested by the Mann–Whitney U test. The median and interquartile range are shown with black bars.

Previous studies have shown that genetic diversity at *env* increases with disease progression in HIV-1-infected individuals in the absence of treatment^24,25^. Hence, to clarify whether elevated genetic diversity associated with disease progression can be observed in entire vCDSs for each sample, we analyzed possible correlations between the magnitude of genetic diversity and two surrogate markers of disease progression, viral load and CD4^+^ T-cell count (Fig. 4 and Supplementary Fig. 1, rs = 0.38, *p* < 0.0001 for viral load and rs = − 0.53, *p* < 0.0001 for CD4^+^ T-cell count). We initially scored intrasample genetic diversity according to the overall mean genetic distances between every pair of nucleotide vCDSs obtained for each sample using the distmat tool implemented in EMBOSS-6.6.0 (https://emboss.sourceforge.net). As shown in Fig. 4, the calculated overall mean genetic distances exhibited statistically significant correlations with both of the disease progression markers. In particular, high genetic distances of HIV-1 were found in samples from patients with ≤ 200 CD4^+^ T-cell counts/μL (*p* < 0.0001). In addition, the two surrogate markers were correlated with the prevalence of nonfixed amino acid mutations at Gag, Pol and Env (Supplementary Fig. 1). These results indicate that nanopore sequencing is useful for estimating the heterogeneity of entire HIV-1 genomes within a sample, as previously shown with *env* sequences^24,25^.

## Discussion

In this study, we developed a new nanopore sequencing-based approach for HIV-1 pangenome sequences using the most recent nanopore flow cell (R10.4.1) and chemistry (V14), which enabled us to determine viral RNA (vRNA) genome sequences in patient plasma with a 15% threshold for minority populations. Retrospective analysis of 106 clinical samples using this system demonstrated that the HIV-1 DR test outcomes were highly concordant with archived test results based on Sanger sequencing-based data (Fig. 3).

Previously, several nanopore sequencing workflows to determine partial sequences of the HIV-1 genome have been reported, especially *pol* for DR tests^26–29^, although our protocol has the advantage of providing comprehensive sequence analyses throughout the long HIV-1 pangenome. Five aspects of this protocol were key in obtaining reliable sequence information for low-abundance mutations via nanopore sequencing. (1) DNA polymerases with high fidelity and low recombination rates were used for RT–PCR/nested PCR amplification. (2) Multiple amplicons per sample were mixed and subjected to library construction for nanopore sequencing. (3) ≥ 100 duplex reads for each sample were obtained using the duplex basecalling system, which is more accurate than the simplex system. (4) The library preparation protocol was customized to avoid unexpected barcode ligation. (5) Bioinformatics processes were devised to reduce possible misalignment in homopolymeric sequences.

In the practical examination of clinical samples with our protocol, the median viral load of the 106 successful amplification cases was approximately 10^5^ copies/mL, whereas that of the 15 unsuccessful cases was approximately 10^4^ copies/mL. The lower limit of the vRNA copy number in plasma samples for RT–PCR/nest PCR amplification was between 10^3^ and 10^4^ copies/mL (Fig. 2). These amplification efficiencies largely depend on the vRNA copy numbers in subtype B, AE and others (Supplementary Fig. 2). However, amplification failure occurred even with a sufficiently high viral load (1,530,000 copies/mL) in the AE sample. According to the sequence information on global epidemic HIV-1 available in the Los Alamos HIV sequence database (https://www.hiv.lanl.gov/), there is one mismatched base at the 3´-end of the primer between the nested PCR (reverse) primer and the AE sample sequence (Supplementary Fig. 3). The mismatched base at the 3´-end of the primer can be ignored because the PrimeSTAR GXL DNA polymerase with exonuclease activity was used in this protocol. Therefore, the other mismatch might have caused failure of amplification by RT–PCR or nested PCR. In addition, two primers used in this study, nested PCR (forward) and RT–PCR (reverse), are identical to the target sequences (+ 2 to + 16 nt position from the 3´-end of primers) in all subtypes of HIV-1 group M, although the RT–PCR (forward) primer has base mismatches (+ 13 and/or + 14 nt position) in CRF02_AG (Supplementary Fig. 3). Therefore, further primer optimization will be needed in cases of suspected infection with these subtypes.

Several studies have reported that DR-associated mutations occur outside drug target genes in HIV-1 genome sequences^30–34^. In addition, Park et al. recently reported the linkage of multidrug cross-resistance mutations between INSTIs and NNRTIs in *pol*^29^. Our nanopore sequencing platform has high potential for identifying mutations throughout the HIV-1 pangenome, which could be used to better understand these genetic linkages. Currently, the error rate of the most recent nanopore sequencing system (approximately 0.1%) is not sufficiently high to determine the pangenome sequence for each DNA molecule, although it is sufficient to identify individual dominant variants even in mixed genetic populations (Supplementary Fig. 4). Recently, Park et al. reported an elegant nanopore sequencing-based workflow that enabled them to obtain accurate single-molecule sequences for the complete *pol* region. Amplicons are prepared by tagging with a unique molecular identifier (UMI) to estimate consensus sequences among the outputted reads sharing each UMI^29^. The combination of such a workflow with our platform might further improve genotyping for minor populations of the HIV-1 pangenome via nanopore sequencing.

In this study, a new nanopore sequencing-based platform for HIV-1 pangenome analysis was developed for HIV-1 DR genotyping. The system is feasible for implementing as powerful comprehensive HIV-1 DR tests in the clinical setting, especially for treatment-naïve patients and treatment-experienced ones with relatively high viral loads. In addition, it is useful for molecular epidemiological surveillance in populations.

## Methods

### Clinical samples

Canonical DR genotyping results determined by Sanger sequencing were obtained from archives of routine laboratory tests of peripheral blood samples from 293 independent HIV-1-infected patients who visited the National Hospital Organization Nagoya Medical Center, Japan, between January 2020 and June 2023. The participants provided written informed consent to participate in the study, which was approved by the Nagoya Medical Center Ethics Committee (approved no. 2010–310) and was conducted according to the principles expressed in the Declaration of Helsinki. Residual plasma samples from 121 of the 293 treatment-naïve patients were subjected to the nanopore sequencing-based protocol in this study.

### In vitro virus preparation

HIV-1 derived from provirus DNAs was prepared in vitro from cell culture as described previously^35^. Briefly, to prepare supernatants containing viruses, either provirus DNA, pNL4-3^36^ or pYK-JRCSF (obtained through the NIH HIV Reagent Program, Division of AIDS, NIAID, NIH)^37^ was transfected into human embryonic kidney 293 T cells using FuGENE HD (Promega). At 12 h after transfection, the culture medium was gently changed with fresh Dulbecco’s modified Eagle’s medium (DMEM) (Sigma–Aldrich) supplemented with 10% fetal bovine serum (FBS) (HyClone), penicillin (100 U/mL) and streptomycin (100 μg/mL) (Thermo Fisher Scientific). The supernatant was harvested at 48 h, clarified by centrifugation and filtration, and used for infection. Viruses were further propagated with the T-cell-derived cell line R5-MaRBLE cells, which stably expresses both CXCR4 and CCR5 coreceptors^38^. The virus supernatant was harvested when cytopathic effects were first observed, and was then clarified by centrifugation and filtration for use in nanopore sequencing analysis.

The amount of vRNA in the supernatants was quantified by RT–digital PCR (RT–dPCR) using a QIAcuity Nanoplate 26 K 24-well instrument and a QIAcuity OneStep Advanced Probe Kit on a QIAcuity dPCR system (QIAGEN) as follows. vRNA was extracted from the culture supernatant (140 µL) using a QIAamp Viral RNA Mini Kit (Qiagen) and eluted with 60 µL of RNase-free distilled water (DW). A total of 5 μL of each vRNA sample was subjected to RT–dPCR analysis in a mixture (total 40 µL) of 4 × OneStep Advanced Probe Master Mix (10 µL), 100 × OneStep Advanced RT Mix (0.4 µL), forward/reverse primers (2.4 µL of 10 µM forward primer [600 nM final] and 1.6 µL of 10 µM reverse primer [400 nM final]) and a fluorescent probe (1 µL of 2 µM probe, i.e., 50 nM at a final concentration). The primer set and probe used were as follows: forward, 5´-AGCCTCAATAAAGCTTGCCTTGA; reverse, 5´-CCCTGTTCGGGCGCCACTGCTAGAG; and probe, 5´-6-FAM (FAM, Carboxyfluorescein)-TCTGGTAACTAGAGATCCCTCAGACC-TAMRA (TAMRA, Carboxytetramethylrhodamine)-3´. The probe targets the poly-A signal loop region in the HIV-1 long-term repeat region. The reaction mixture was incubated as follows: RT extension (40 min at 50 °C), denaturation (2 min at 95 °C) and 40 cycles of denaturation (5 s at 95 °C) and annealing-extension (30 s at 60 °C). Based on the vRNA concentration determined by RT–dPCR, HIV-1 NL4-3 and HIV-1 JRCSF virus samples were diluted to approximately 20,000 copies/mL with fresh culture medium for RNA extraction and nanopore sequencing.

### vRNA extraction and DNA preparation

vRNA was extracted from 140 µL of patient plasma using a QIAamp Viral RNA Mini Kit (Qiagen) and eluted with 60 µL of RNase-free DW according to the manufacturer’s instructions. DNA fragments of HIV-1 pangenomes were obtained from the RNA by one-step RT–PCR followed by nested PCR as previously described^4^ with minor modifications. The sequences of the forward and reverse primers for RT–PCR were 5´-CTCTCTCGACGCAGGACTCGGCTTG (corresponding to positions 681–705 nt in the reference HXB2 sequence [accession #K03455] in the Los Alamos HIV Sequence Database [https://www.hiv.lanl.gov/]) and 5´-CACTCAAGGCAAGCTTTATTGAGGC (positions 9631–9607 nt). The primer sequences for the nested PCR were 5´-TTTGACTAGCGGAGGCTAGAAGGAGA (761–786 nt) and 5´- GGTCTAACCAGAGAGACCCAGTACAG (9557–9532 nt). The primers were designed to target highly conserved regions across multiple major subtypes of HIV-1 according to publicly available sequences. All the DNA primers used were purchased from Fasmac Co. To minimize the possibility of false-positive detection of mutations, RT–PCR followed by nested PCR was performed independently three or more times per sample^13,14^.

RT–PCR was performed with a PrimeScript II High Fidelity One Step RT–PCR Kit (Takara Bio) in a total volume of 25 μL containing 2 × One Step High Fidelity Buffer (12.5 µL), RT–PCR primers (1 µL of an equimolar mixture of each 2.5 µM primer, i.e., 0.1 μM each at final in a reaction), PrimeScript II RT Enzyme Mix (0.5 µL), PrimeSTAR GXL (2 µL) and vRNA (3 µL). vRNA extracted from individual samples with viral loads greater than 50,000–100,000 copies/mL was diluted to less than 100,000 copies/mL with poly-A RNA (20 μg/mL) (Merck) in DW because a high concentration of vRNA tends to reduce amplification efficiency. The RT–PCR conditions were as follows: (1) 15 min at 45 °C for reverse transcription, (2) 2 min at 94 °C for denaturation and DNA polymerase activation, and (3) 45 cycles of denaturation (10 s at 98 °C) and annealing-elongation (100 s at 68 °C).

Nested PCR was performed with PrimeSTAR GXL DNA polymerase (Takara Bio) in a total volume of 25 μL: 5 × PrimeSTAR GXL Buffer (5 µL), dNTPs (2 µL of an equimolar mixture of each 2.5 mM dNTP, i.e., final concentration of 200 µM each), nested PCR primers (1 µL of an equimolar mixture of each 2.5 µM primer), PrimeSTAR DNA GXL polymerase (1 µL) and the RT–PCR product (2 µL). Nested PCR was performed in 35 cycles of denaturation (10 s at 98 °C) and annealing-extension (90 s at 68 °C).

The sizes of the resulting nested PCR products were analyzed by 0.7% agarose gel electrophoresis with ethidium bromide staining and/or Bioanalyzer DNA analysis with a DNA 12,000 Kit (Agilent). The products were expected to appear as a single band (~ 8.8 k base pairs) in the electrophoresis. When single bands of the expected size were obtained in two or more reactions per sample, the PCR products were mixed and stored at − 80 °C for further use. The pooled products were purified with a MultiScreen HTS PCR96 filter plate (Merck). The concentration of the eluate with DW (25 µL) was measured with a Qubit dsDNA BR assay kit (Thermo Fisher Scientific) and adjusted to 100 fmol in 15 μL with DW.

### Library preparation

The procedure for multiplexed library preparation was slightly modified from the manufacturer’s protocol of “Ligation sequencing amplicons—Native Barcoding Kit 24 V14 (SQK-NBD114.24)” (ONT); specifically, to avoid unexpected ligation of PCR-amplified DNA and nonspecific barcodes, the DNA products were pooled after adaptor ligation, whereas the manufacturer protocol suggests to pool the DNA samples before adaptor ligation.

The DNA ends of each nested PCR product were first repaired and dA-tailed with the NEBNext Ultra II End Repair/dA-tailing Module (New England Biolabs [NEB]). We mixed 15.0 μL of the PCR product (100 fmol), Ultra II End-Prep Reaction Buffer (2.1 µL), and Ultra II End-Prep Enzyme Mix (0.9 µL) and then incubated the mixture for 5 min at 20 °C followed by 5 min at 65 °C. The end-repaired products were purified with a MultiScreen HTS PCR96 filter plate and eluted with DW (25 µL). Subsequently, barcodes and adaptors were ligated onto each end-repaired product via either a one- or two-step reaction. The one-step reaction is less time-consuming and less efficient than the two-step one. For the one-step reaction, 25 μL of the end-repaired DNA was mixed with 1.5 μL of a native barcode (Native Barcoding Kit 24 V14 [SQK-NBD114.24], ONT), 1.5 μL of the native adaptor (ONT) and 28 μL of the Blunt/TA Ligase Master Mix (NEB). The mixture was incubated for 20 min at 25 °C. Alternatively, for the two-step reaction, 7 μL of the end-repaired DNA product was mixed with a native barcode (ONT) (1 µL) and Blunt/TA Ligase Master Mix (NEB) (8 µL). After a 20-min incubation at 25 °C, the reaction products were purified with a MultiScreen HTS PCR96 filter plate and eluted with DW (15 µL). Adaptor ligation was performed using the NEBNext Quick Ligation Module (NEB) with barcoded DNA (6.8 µL), 5 × Quick Ligation buffer (2 µL), Quick T4 ligase (1 µL) and the native adaptor (ONT) (0.2 µL) for 20 min at 25 °C. The adaptor ligation products were separately purified with AMPure XP beads (Beckman Coulter) (22.4 μL and 9 μL for one- and two-step reactions, respectively). After two washes with 60 μL of long fragment buffer (LFB) (ONT), the products were eluted with elution buffer (12 µL) (ONT, cat# SQK-NBD114.24). The concentration of each product was quantified with a Qubit dsDNA HS assay kit (Thermo Fisher Scientific) and adjusted to 16 fmol in 12 μL using DW. More than two products (to eight) were pooled to a 12 μL library for nanopore sequencing.

### Nanopore sequencing

The flow cell priming mix with UltraPure BSA (Thermo Fisher Scientific) was used according to the protocol of the Ligation Sequencing Amplicons—Native Barcoding Kit 24 V14 (SQK-NBD114.24)” (ONT). The priming mixture was loaded onto an R10.4.1 MinION flow cell (FLO-MIN114, ONT). Nanopore sequencing was performed using the MinION Mk1C device (ONT) for a maximum of 24 h with a new flow cell or for 48 h with a flow cell once the device was reprimed with the Wash Kit (EXP-WSH004, ONT). When multiple runs were performed with a flow cell, the flow cell was washed between runs, and different sets of barcodes were used for each run as an additional guard against sample cross-contamination.

### Base-calling and read selection

DNA bases were called to obtain simplex and duplex reads by the dorado program ver. 0.3.0 (https://github.com/nanoporetech/dorado) in SUP mode (model dna_r10.4.1_e8.2_400bps_sup@v4.2.0) on a Linux server in our laboratory. For the duplex read base calling, the additional option “–min-qscore 10” was used. The obtained raw reads were filtered by length with the NanoFilt program^39^ as reported previously^4^. The raw duplex reads were further selected as follows. First, to improve alignment quality, an individual reference sequence for each sample was generated. Briefly, the reads were aligned onto a pangenomic region of the HXB2 sequence (790–9719 nt) by the minimap2 program (ver. 2.24) with the option “-a -x map-ont -A 2 -O 24,24 -E 2,2 –secondary = no” to avoid ambiguous mapping and unnecessary gap opening. When an aligned read contained a “ZZ–YXXXX (X ≠ Y, Y ≠ Z)” sequence context, in which [X, Y, Z] and [–] represent given bases and a deletion, respectively, the context was corrected to “ZZY–XXXX” through our in-house program. This correction was added because in nanopore sequencing, one-base deletion errors generally occur more frequently in long homopolymer regions than in nonhomopolymeric regions^12^. Next, a consensus sequence generated based on the aligned reads was compared with the HXB2 reference sequence. The discordant bases between the consensus and reference at the corresponding genome positions were converted on the HXB2 sequence template, and a sample-specific reference sequence was generated. Moreover, the reads were aligned onto the sample-specific reference sequence by minimap2 with the same option. Each “ZZ–YXXXX” context within a read was corrected in the manner described above. Finally, at least 100 raw duplex reads containing the entire vCDS of the HIV-1 genome (790–9417 bp between *gag* and *nef* in HXB2) were selected and obtained from each sample for further analyses. All 100 raw duplex reads containing the entire vCDSs of the HIV-1 genome (positions 790–9417 nt between *gag* and *nef* in HXB2) were selected and obtained from each sample for further analyses. Samples with less than 100 raw reads were excluded from the subsequent analyses. Of note, in this study, approximately 30,000 raw simplex reads were required to obtain more than 100 reads with the entire vCDSs.

### Sequence data analyses

In our analytical protocol, we have used the in-house PERL scripts and publicly available programs such as dorado (https://github.com/nanoporetech/dorado [the version 0.3.0 was used for the test]), samtools (http://www.htslib.org/ [the version 1.1]), NanoFilt (https://github.com/wdecoster/nanofilt [the version 2.8.0])^39^, and minimap2 (https://github.com/lh3/minimap2 [the version 2.24]). The PERL scripts are available in GitHub (https://github.com/odehir/ONT-Kit14_HIV1). In addition, examples of Shell scripts for automatic program execution (DR-test_SUP.Q10.dorado.w-barcode.sh, extract-gene.na.all.sh and extract-gene.aa.all.sh) are also available in the GitHub site. Because the exemplified Shell scripts are written at the specific settings of “minit” as the user name and “/home/minit/nanopore/tools/” as the location of the scripts on a Linux environment, customization of paths to the programs and scripts may be required for a user who adopts a different computer environment. When the main Shell script (DR-test_SUP.Q10.dorado.w-barcode.sh) is placed on /home/minit/nanopore/tools/, it may work using the following command: /home/minit/nanopore/tools/DR-test_SUP.Q10.dorado.w-barcode.sh (full path to “pod5” directory) (full path to working directory).

In this study, we performed base calling and sequence data analyses on a Linux server (Ubuntu 20.04) with NVIDIA RTX A5000 and the Intel Core i9-10980XE (18 CPU cores) in our laboratory. The rate-limiting process was the base calling with the SUP mode. We have confirmed that the scripts work on a Mac OS computer with M2 chip and Ubuntu 20.04 on the Windows Subsystem for Linux 2 in a Windows 11 computer with NVIDIA GeForce GTX 1650 Ti, upon individual customization of the Shell scripts for each environment. However, compared to the Linux server, the other two computers displayed quite low analytical speeds, especially at the base calling.

### Detection of mutations

Individual viral gene sequences were aligned from duplex reads covering the entire vCDS based on the HIV-1 HXB2 reference sequence. The intrasample occupancies of the nucleotide and amino acid mutations at each position were analyzed by in-house programs. HIV-1 DR mutations were scored according to the ANRS algorithm (V34, November 2023) (https://hivfrenchresistance.org/). The coreceptor usage of HIV-1 for each sequencing read was predicted by submitting the V3 region sequences to the Geno2pheno[coreceptor] server (https://coreceptor.geno2pheno.org/) with the geno2pheno-C_NGS-Sanger algorithm^20^.

### Determination of HIV-1 subtypes

Using the entire vCDS, clusters were first estimated using the Sumaclust program (ver. 1.0.36)^40^ with the option “-t 0.99”. Subtyping was achieved with the jpHMM-HIV (ver. Mar 2015)^15^ using vCDSs located at the centers of clusters with > 1% of intrasample occupancies.

### Phylogenetic tree analysis

Phylogenetic tree analysis of the entire vCDS from duplex reads was performed by multiple alignment including the HXB2 reference sequence, using MAFFT (ver. 7.372)^41^. A neighbor-joining tree was constructed with rapidNJ ver. 2.2.2 (https://birc.au.dk/software/rapidnj) by 500 replicates of bootstrap tests. The tree results were drawn with FigTree ver. 1.4.4 (http://tree.bio.ed.ac.uk/software/figtree/).

### Analysis of coexisting mutations

Nonrandom linkage between mutations was assessed on the basis of the *r*^2^ score of linkage disequilibrium^42^. Each pair of coexisting mutations with *r*^2^ > 0.5 was screened^42^ and a positive or negative correlation was assessed. In the event of a mixed infection patient, a relatively stringent *r*^2^ score (> 0.9) was set to detect coexisting mutations with positively correlated linkages in individual read sequences of the HIV-1 pangenome.

### Estimation of genetic distance

Genetic diversity among vCDSs within duplex reads per sample was analyzed by multiple alignments using MAFFT program (ver. 7.372)^41^. The uncorrected distance matrix was calculated by the distmat tool implemented in EMBOSS-6.6.0 (https://emboss.sourceforge.net/), and averages of the distances were obtained. These calculated distances represent substitution values per 100 bases as genetic distances among nanopore-sequencing reads.

### Statistical tests

The non-parametric multiple comparison test, Mann–Whitney U test and Spearman’s rank-order correlation were performed for nonparametric tests using Prism 9 software (GraphPad).

### Supplementary Information

Supplementary Information.

## Data Availability

The sequencing data obtained in this study are available in the DNA Data Bank of Japan (DDBJ) Sequenced Read Archive (DRA) under BioProject accession ID PRJDB17699, which includes duplex reads of NL4-3 and JRCSF (DRA run accession IDs DRR537713 and DRR537714) and HIV-1 from 106 clinical samples (DRR537715-DRR537820).

## Code availability

The in-house programs are accessible in GitHub (https://github.com/odehir/ONT-Kit14_HIV1).
