# Supplementary material for: Population-based nanopore sequencing of the HIV-1 pangenome to identify drug resistance mutations
Source: Sci Rep. 2024 May 27;14:12099. doi: 10.1038/s41598-024-63054-3 (PMC11130118; doi:10.1038/s41598-024-63054-3)
Supplement: Supplementary file 1 — Supplementary Information. [file 41598_2024_63054_MOESM1_ESM.pdf]

## Supplementary Methods

### Sanger sequencing for HIV-1 drug resistance (DR) genotyping

For DR genotyping, we determined the nucleotide sequences of four HIV-1 target regions by Sanger sequencing: *gag matrix* (positions 790-1,185 nt according to the position numbering of the reference HXB2 strain), *pol protease (PR)-reverse transcriptase (RT)* (2,253-3,269 nt), *pol integrase (IN)* (4,230-5,093 nt) and *env c2c5* (6,896-7,652 nt)[4,16-19,43-47]. Briefly, we first performed RT–PCR with the PrimeScript II High Fidelity One Step RT–PCR Kit (Takara Bio) under the following conditions: reverse transcription (10 min at 45°C), denaturation (2 min at 94°C) and 40 cycles of denaturation (10 sec at 98°C), annealing (10 sec at 52°C) and extension (15 sec for the *pol PR-RT* and *pol IN* or 10 sec for the *gag matrix* and *env c2c5* at 68°C). Nested PCR was subsequently performed with PrimeSTAR GXL DNA Polymerase (Takara Bio) as follows: 40 cycles of denaturation (10 sec at 98°C), annealing (10 sec at 55°C) and extension (15 sec for *pol PR-RT* and *pol IN* or 10 sec for the *gag matrix* and *env c2c5* at 68°C). PCR products were purified with MultiScreen HTS PCR96 filter plates (Merck). The final DNA products were subjected to Sanger sequencing with a BigDye Cycle Sequencing Kit (Thermo Fisher Scientific) in a 3730 DNA Analyzer (Thermo Fisher Scientific). PCR was performed according to the manufacturer's protocol as follows: 1) denaturation and Taq DNA polymerase activation (15 sec at 96°C); 2) 30 cycles of denaturation (10 sec at 96°C) and annealing-extension (50 sec at 60°C); and 3) 4°C hold. All the oligonucleotide primers used in the DR genotyping tests are listed in Supplementary Table S1. For the *matrix* and *env c2c5* regions, only a major peak base at each nucleotide position was called, because these two regions are more prone to heterogeneity than the *pol* region[3]. For *pol*, both a major peak bases and low-abundance bases were read as mixed bases according to the sequencing electropherogram data when these bases were detected in both the forward and reverse strand sequences. Sequencing of *c2c5* was performed on three independent amplicons per sample to analyze its heterogeneity.

For analysis of *gag capsid* (1,186-1,878 nt) sequences, we developed an additional protocol

based on Sanger sequencing. Briefly, DNA fragments of the *gag-PR* region were amplified by RT–PCR using the PrimeScript II High Fidelity One Step RT–PCR Kit. The RT–PCR mixture was incubated as follows: 1) RT extension (10 min at 45°C); 2) denaturation (2 min at 94°C); and 3) 40 cycles of denaturation (10 sec at 98°C), annealing (10 sec at 56°C) and extension (45 sec at 68°C). The first-round PCR products were then subjected to nested PCR with PrimeSTAR GXL DNA polymerase under the following conditions: 30 cycles of denaturation (10 sec at 98°C), annealing (10 sec at 58°C) and extension (30 sec at 68°C). The nested PCR products were then purified using a MultiScreen HTS PCR96 filter plate. The *capsid* sequences were determined by Sanger sequencing in the same manner as described above for the DR genotyping test. The primers for RT–PCR, nested PCR and Sanger sequencing are listed in in Supplementary Table S1. For analysis of DR mutations in the *capsid* region, we selected dominant nucleotide bases as well as low-abundance bases in the electropherograms when mixed bases were observed in both the forward and reverse strands.

**Supplementary Table 1.** The primer sets for DR genotyping tests by Sanger sequencing

| Target gene | Purpose | F or R* | Sequence (5′ to 3′) | Positions (5′-to-3′)† |
|---|---|---|---|---|
| *pol PR-RT* | RT–PCR | F | AAGGGCTGTTGGAAATGTGG | 2,020-2,039 |
| | | R | CCCACTCAGGAATCCAGGT | 3,792-3,774 |
| | Nested PCR | F | GAAAGGAAGGACACCAAATGA | 2,039-2,059 |
| | | R | CTCATTCTTGCATATTTTCCTGTT | 3,622-3,599 |
| | Sequencing | F | TCACTCTTTGGCAACGACCC | 2,260-2,279 |
| | | F | TTAAAGCCAGGAATGGATG | 2,583-2,601 |
| | | F | ACAGAAATGGAAAAGGAAGG | 2,664-2,683 |
| | | F | ATACTGCATTTACCATACC | 2,929-2,947 |
| | | R | CTGGCTTTAATTTTACTGGTA | 2,592-2,572 |
| | | R | AGTATTGTATGGATTTTCAGGC | 2,723-2,702 |
| | | R | TGATCCTTTCCATCCCTG | 3,017-3,000 |
| | | R | CTCATTCTTGCATATTTTCCTGTT | 3,622-3,599 |
| *pol IN* | RT–PCR | F | CAGACTCACAATATGCATTAGG | 4,039-4,060 |
| | | R | CCTGTATGCAGACCCCAATATG | 5,264-5,243 |
| | Nested PCR | F | CTGGCATGGGTACCAGCACACAA | 4,146-4,168 |
| | | R | TAGTGGGATGTGTACTTCTGAAC | 5,217-5,195 |
| | Sequencing | F | CATGGGTACCAGCACACAAAG | 4,150-4,170 |
| | | F | ATGCATGGACAAGTAGACTG | 4,377-4,396 |
| | | R | TGAATACTGCCATTTGTACTG | 4,773-4,753 |
| | | R | TGGGATGTGTACTTCTGAACTTA | 5,214-5,192 |
| *gag matrix* | RT–PCR | F | ATCTCTAGCAGTGGCGCCCGAACAG | 625-649 |
| | | R | CTGATAATGCTGAAAACATGGGTAT | 1,318-1,294 |
| | Nested PCR | F | CTCTCTCGACGCAGGACTCGGCTTG | 681-705 |
| | | R | CCCATGCATTCAAAGTTCTAGGTGA | 1,255-1,231 |
| | Sequencing | F | CTCTCTCGACGCAGGACTCGGCTTG | 681-705 |
| | | F | GACTAGCGGAGGCTAGAAGG | 764-783 |
| | | R | CCCATGCATTCAAAGTTCTAGGTGA | 1,255-1,231 |
| *env c2c5* | RT–PCR | F | GAGCCAATTCCCATACATTATTGT | 6,855-6,878 |
| | | R | GCCCATAGTGCTTCCTGCTGCTCCCAAGAACC | 7,817-7,786 |
| | Nested PCR | F | TTATTGTGCCCCAGCTGGTTTTGC | 6,872-6,895 |
| | | R | TATATAATTCACTTCTCCAATTGTC | 7,677-7,653 |
| | Sequencing | F | TTATTGTGCCCCAGCTGGTTTTGC | 6,872-6,895 |
| | | R | CAGTAGAAAAATTCYCCTCYACAA | 7,378-7,355 |
| | | R | TATATAATTCACTTCTCCAATTGTC | 7,677-7,653 |
| *gag capsid* | RT–PCR | F | ATCTCTAGCAGTGGCGCCCGAACAG | 625-649 |
| | | R | TATGGATTTTCAGGCCCAATTTTTGA | 2,716-2,691 |
| | Nested PCR | F | CTCTCTCGACGCAGGACTCGGCTTG | 681-705 |
| | | R | ACTTTTGGGCCATCCATTCC | 2,611-2,592 |
| | Sequencing | F | CAGCATTATCAGAAGGAGCCACCCC | 1,307-1,331 |
| | | F | ATCAATGAGGAAGCTGCAGAATGGG | 1,402-1,426 |
| | | R | GTTCCTGCTATGTCACTTCCC | 1,505-1,485 |
| | | R | ACTCCCTGACATGCTGTCATCATTTC | 1,847-1,822 |

* F and R denote forward and reverse primers, respectively.

† The numbers represent the nucleotide positions in the HXB2 reference sequence.

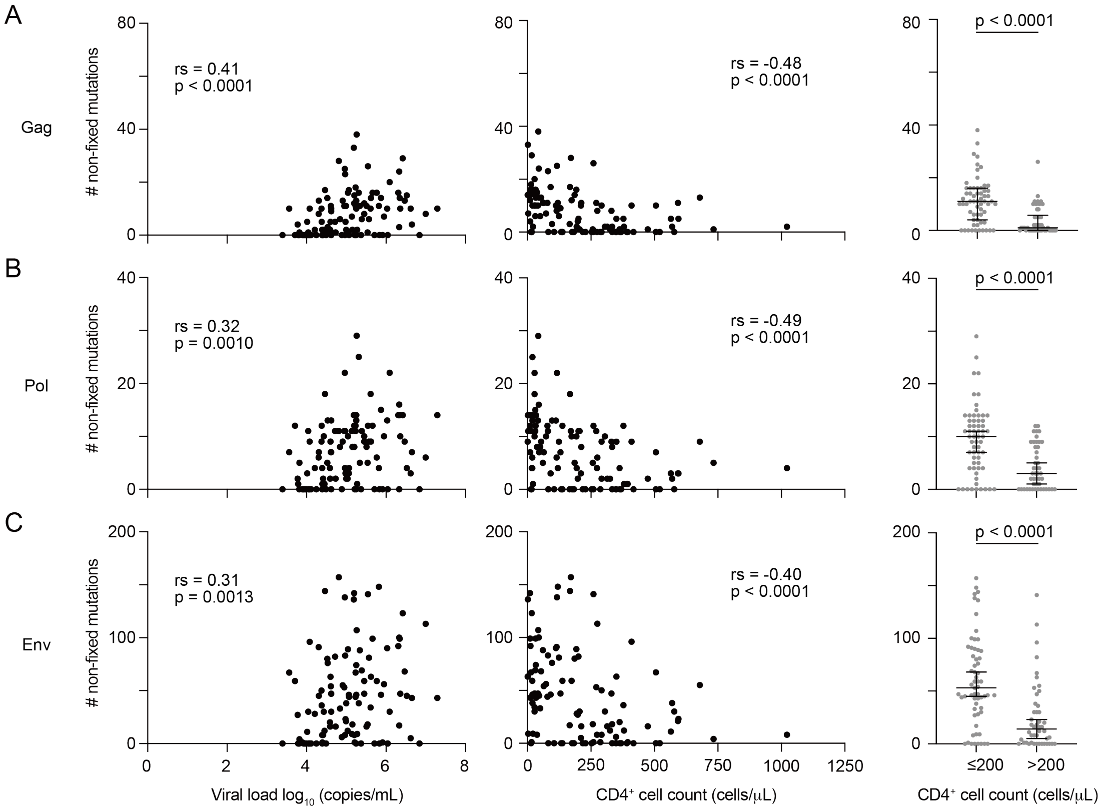


**Supplementary Figure 1.** The relationship of genetic diversity with two surrogate markers for HIV-1 disease progression: viral load $\log_{10}$ and $CD4^+$ T-cell count. The genetic diversity for each sample was assessed by plotting the number of nonfixed amino acid mutations (15-90% of intrasample prevelence) in Gag (**A**), Pol (**B**) and Env (**C**). Correlations of genetic diversity with viral load (left) or $CD4^+$ T-cell count (middle) were evaluated with the nonparametric Spearman's rank-order test. In the right panels, the genetic diversity is compared between two groups of patient samples with $CD4^+$ T-cell counts of ≤200 and >200 cells/μL. Mann–Whitney U tests were used to analyze the statistical significance of the differences between groups. The median and IQR are shown with black bars.

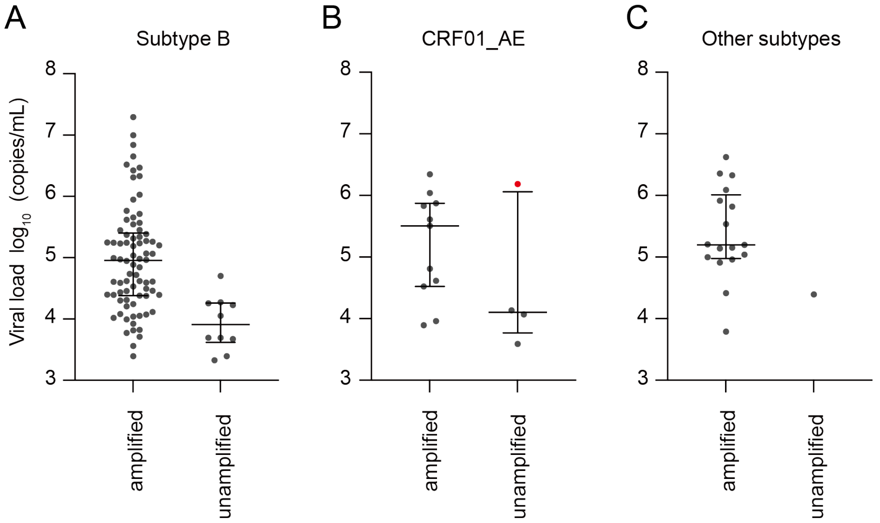


**Supplementary Figure 2.** Limits of HIV-1 pangenome amplification by RT–PCR and nested PCR. Lower limit of the viral RNA concentration in plasma from treatment-naïve patients. Amplification tests were performed in quadruplicate using 121 samples with various viral loads (copies/μL). The $\log_{10}$-transformed viral loads are plotted with gray dots in two groups: successful amplification (amplified) and no amplification in any of the quadruple reactions (unamplified). The median and interquartile range (IQR) are shown with black bars. The plots are separately shown for HIV-1 subtype B (n=88) (**A**), CRF01_AE (n=15) (**B**) and other subtypes (n=18) (**C**). The one CRF01_AE sample of which amplification was failed despite its high viral load is shown as a red dot.

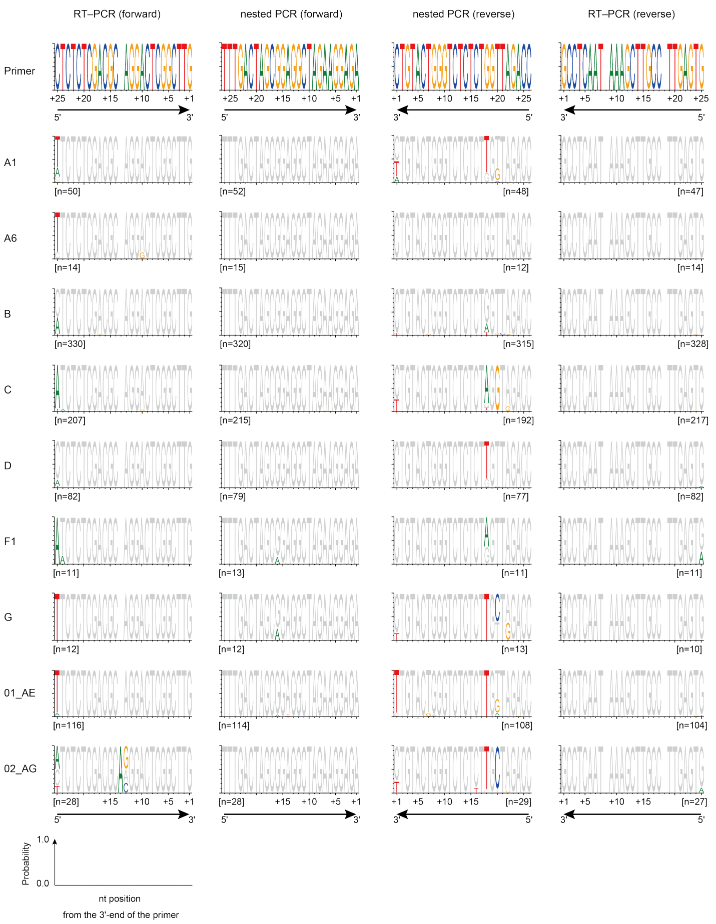


**Supplementary Figure 3.** Variation in HIV-1 sequences targeted by primers used for RT–PCR and nested PCR in this study. A total of 1,231 HIV-1-genome sequences covering 681-9,631 nt (nt positions are based on the HXB2 reference) were extracted from the Los Alamos HIV sequence database (https://www.hiv.lanl.gov/; downloaded in December 2023) with the two criteria set as follows: 1) the "one sequence/patient" option and 2) no restriction on the country of sample collection. For a given subtype, we grouped representative sequences that were shared among 2 or more samples for every primer-targeting sequence. Only subtypes for which ≥10 representative sequences were obtained from the database are shown. The representative sequences for each subtype were subjected to multiple sequence alignment by MEGAX[48]. The conservation rate at each position was analyzed using WebLogo (ver. 3.5.0)[49], and probability values are shown on the ***y***-axis. The direction of each primer is indicated with a black arrow. The nucleotide positions of the primers are numbered starting from the 3´-end. Nucleotides that matched and mismatched to those in the reference are indicated in light gray and colored letters, respectively.

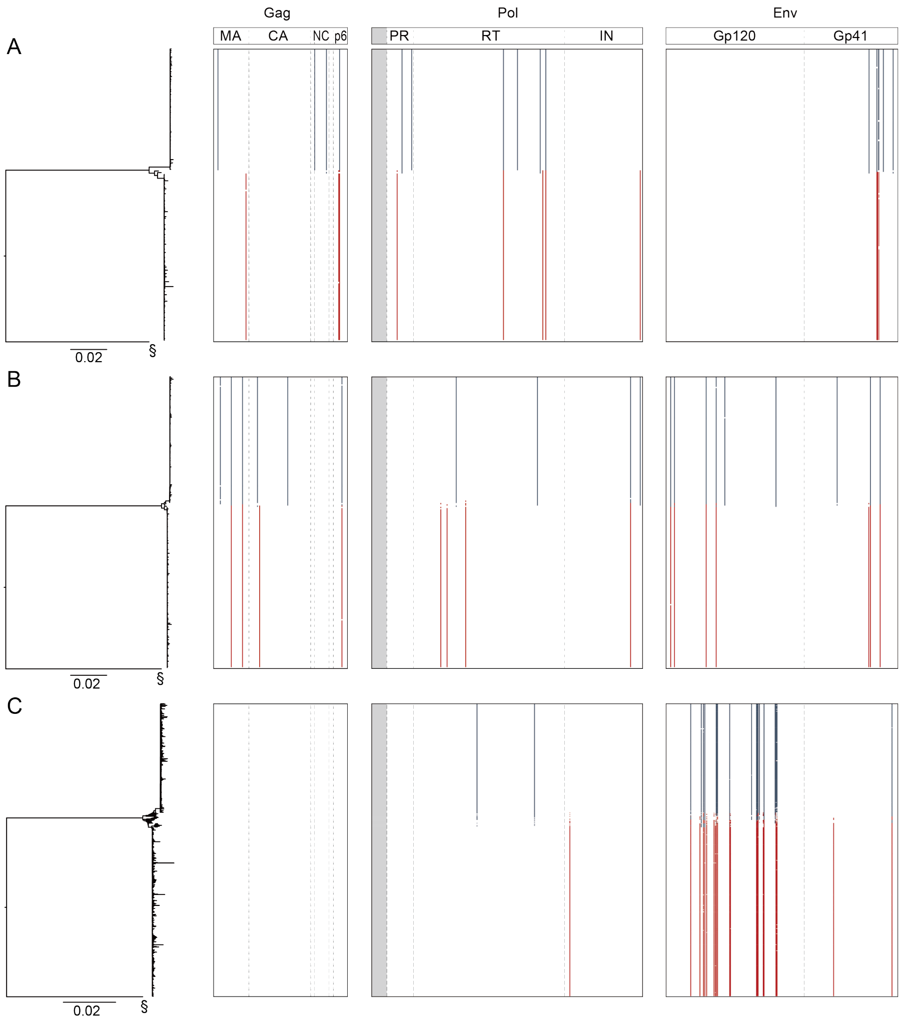


**Supplementary Figure 4.** Two closely related variant clusters bearing different sets of mutations across major polyproteins (Gag, Pol, and Env) were detected concomitantly in three patients. Phylogenetic trees based on entire vCDSs in duplex reads of nanopore sequencing are shown on the left side. The branches of the HXB2 reference are indicated with "§". The trees were drawn with FigTree (ver. 1.4.4) (http://tree.bio.ed.ac.uk/software/figtree/). Within amino acid mutations in the respective sequences, coexisting nonfixed mutations with highly positively correlated linkages within each cluster are shown on the right side. These mutations are highlighted in blue or red bars at the amino acid positions of the polyproteins. Amino acid mutations at the trans-frame region named TFR or p6* in Pol (gray backgrounds) are not shown. The three cases are the samples with viral swarms of CRF01_AE (*A*), subtype C (*B*), and subtype B (*C*).

**References for Supplemental Materials**


43 Parbie, P. K. *et al.* High-level resistance to non-nucleos(t)ide reverse transcriptase inhibitor based first-line antiretroviral therapy in Ghana; A 2017 study. *Front Microbiol* **13**, 973771, doi:10.3389/fmicb.2022.973771 (2022).

44 Gatanaga, H. *et al.* Drug-resistant HIV-1 prevalence in patients newly diagnosed with HIV/AIDS in Japan. *Antiviral Res* **75**, 75-82, doi:10.1016/j.antiviral.2006.11.012 (2007).

45 Hattori, J. *et al.* Characteristics of Transmitted Drug-Resistant HIV-1 in Recently Infected Treatment-Naive Patients in Japan. *J Acquir Immune Defic Syndr* **71**, 367-373, doi:10.1097/QAI.0000000000000861 (2016).

46 Shiino, T., Hachiya, A., Hattori, J., Sugiura, W. & Yoshimura, K. Nation-Wide Viral Sequence Analysis of HIV-1 Subtype B Epidemic in 2003-2012 Revealed a Contribution of Men Who Have Sex With Men to the Transmission Cluster Formation and Growth in Japan. *Front Reprod Health* **2**, 531212, doi:10.3389/frph.2020.531212 (2020).

47 Shiino, T. *et al.* Phylodynamic analysis reveals CRF01_AE dissemination between Japan and neighboring Asian countries and the role of intravenous drug use in transmission. *PLoS One* **9**, e102633, doi:10.1371/journal.pone.0102633 (2014).

48 Kumar, S., Stecher, G., Li, M., Knyaz, C. & Tamura, K. MEGA X: Molecular Evolutionary Genetics Analysis across Computing Platforms. *Mol Biol Evol* **35**, 1547-1549, doi:10.1093/molbev/msy096 (2018).

49 Crooks, G. E., Hon, G., Chandonia, J. M. & Brenner, S. E. WebLogo: a sequence logo generator. *Genome Res* **14**, 1188-1190, doi:10.1101/gr.849004 (2004).
